# Increasing buffering capacity enhances rumen fermentation characteristics and alters rumen microbiota composition of high-concentrate fed Hanwoo steers

**DOI:** 10.1038/s41598-022-24777-3

**Published:** 2022-12-01

**Authors:** Sonny C. Ramos, Seon Ho Kim, Chang Dae Jeong, Lovelia L. Mamuad, A-Rang Son, Seung Ha Kang, Yong Il Cho, Tae Gyu Kim, Jin Sung Lee, Kwang Keun Cho, Sung Sill Lee, Sang Suk Lee

**Affiliations:** 1 Ruminant Nutrition and Anaerobe Laboratory, Department of Animal Science and Technology, Sunchon National University, 413 Jungangno, Jeonnam, Suncheon, 57922 Republic of Korea; 2 Faculty of Medicine, The University of Queensland Diamantina Institute, Brisbane, Australia; 3 Animal Disease and Diagnostic Laboratory, Department of Animal Science and Technology, Sunchon National University, 413 Jungangno, Jeonnam, Suncheon, 57922 Republic of Korea; 4 Rupromin Balance™, 5th. Bonsol Blg. 445, Teheran-ro, Gangnam-gu, Seoul, 06158 Republic of Korea; 5 Department of Animal Resources Technology, Gyeongnam National University of Science and Technology, Jinju, 52725 Republic of Korea; 6 Institute of Agriculture and Life Science and University-Centered Labs, Gyeongsang National University, Jinju, 52828 Republic of Korea

**Keywords:** Animal physiology, Microbiology

## Abstract

The buffering capacity of buffer agents and their effects on in vitro and in vivo rumen fermentation characteristics, and bacterial composition of a high-concentrate fed Hanwoo steers were investigated in this study. Treatments were comprised of CON (no buffer added), BC_0.3%_ (low buffering capacity, 0.3% buffer), BC_0.5%_ (medium buffering capacity, 0.5% buffer), and BC_0.9%_ (high buffering capacity, 0.9% buffer). Four Hanwoo steers in a 4 × 4 Latin square design were used for the in vivo trial to assess the effect of treatments. Results on in vitro experiment showed that buffering capacity, pH, and ammonia–nitrogen concentration (NH_3_-N) were significantly higher in BC_0.9%_ and BC_0.5%_ than the other treatments after 24 h incubation. Individual and total volatile fatty acids (VFA) concentration of CON were lowest compared to treatment groups. Meanwhile, in vivo experiment revealed that Bacteroidetes were dominant for all treatments followed by Firmicutes and Proteobacteria. The abundances of *Barnesiella intestinihominis, Treponema porcinum,* and *Vibrio marisflavi* were relatively highest under BC_0.9%,_ *Ruminoccocus bromii* and *Succiniclasticum ruminis* under BC_0.5%_, and *Bacteroides massiliensis* under BC_0.3%._ The normalized data of relative abundance of observed OTUs’ representative families have grouped the CON with BC_0.3%_ in the same cluster, whereas BC_0.5%_ and BC_0.9%_ were clustered separately which indicates the effect of varying buffering capacity of buffer agents. Principal coordinate analysis (PCoA) on unweighted UniFrac distances revealed close similarity of bacterial community structures within and between treatments and control, in which BC_0.9%_ and BC_0.3%_ groups showed dispersed community distribution. Overall, increasing the buffering capacity by supplementation of BC_0.5%_ and and BC_0.9%_ buffer agents enhanced rumen fermentation characteristics and altered the rumen bacterial community, which could help prevent ruminal acidosis during a high-concentrate diet.

## Introduction

The rumen is a complex microbial ecosystem harboring compartment, hosting abundant bacteria^1,2^ and constitutes an effective animal-microbe mutualism system^3,4^. Ruminants depend on rumen microbes for structural carbohydrates degradation, and volatile fatty acids (VFA) and microbial protein synthesis as major sources of energy and protein^5^. Energy and essential nutrients are obtained by ruminants through a complicated symbiotic relationship with the rumen microbiome^6^. A high forage diet is usually switched to a high concentrate diet to improve the productivity of the ruminants; however, it alters the rumen ecosystem due to high non-structural carbohydrate level^6^. High grain or concentrate diets alter the rumen microbial ecosystem which increases the rate of ruminal fermentation of short-chain fatty acids (SCFAs), and thus promote rapid growth in the ruminant production system. Bacterial community alterations can also affect the health of the host animal^7^. With the increase of SCFAs in high grain diet, it can exceed the ability of rumen fluid buffers to maintain an optimal pH by exceeding the rate of absorption in the rumen wall, which could, later on, lead to ruminal acidosis.

High-concentrate diet could be attributed in the reduction of Firmicutes in the rumen, as it was previously reported to induce apoptosis and cytolysis^8,9^. High-concentrate diet can also enhance the growth of lactic acid utilizers like *Megasphaera elsdenii*, *Selenomonas ruminantium*, and *Veillonella parvula*, which, in turn, could drastically reduce the abundance of fiber-degrading bacteria such as *Fibrobacter succinogenes* and *Ruminococcus* spp.^6^. Although *Ruminococcus* spp. are well-known cellulolytic bacteria, several species are capable of fermenting starch such as *R. bromii*^10–12^. Feeding of highly fermentable diets is the current practices in high producing beef to increase growth rates, but it causes microbial disturbances resulting to digestive disorders such as ruminal acidosis^13^. The rapid fermentation of non-structural carbohydrates resulted in the accumulation of volatile fatty acid and lactic acid in the rumen causing a drastic decrease in pH^14^. Hence, the use of buffering agents could be useful to resist changes in rumen pH whenever cattle are being fed with high concentrate, low forage, fermented and fine-chopped forage^15^. Compounds that increase the buffering capacity of ruminal fluid help maintain a more stable ruminal pH. It can also direct neutralization of VFA especially during a diet or experimental challenge that could induce ruminal acidosis^13,14^. Rumen buffering could avert the sudden decrease in pH, thus could enhance rumen microbial growth, activity and diversity, microbial protein synthesis, and fermentation end product^16^. Buffering capacity (BC) is then referred to as the number of moles of H^+^ that should be added to a 1L solution to decrease pH by 1 unit^17^. Weak acids and bases are known to provide better buffering in comparison to strong acids and bases because of the equilibrium establishment between the acid and the conjugate base^18^.

Various studies have reported that adding a buffer solution, such as sodium bicarbonate (NaHCO_3_) with magnesium oxide (MgO) increased dry matter intake when corn silage was the sole or major source of forage in the diet^19^. NaHCO_3_ is commonly used in preventing ruminal acidosis because it provides a natural buffer; however, its high solubility limits the buffering activity against acidic conditions^20^. Le Ruyet and Tucker^21^ proved that NaHCO_3_ had high BC in an in vitro study. It contained 26% more of the actively buffering CO_3_ portion of the molecule, which is essential to neutralize acid regardless if ruminal fluid is saturated with CO_2_. In addition, MgO functions efficiently in combination with NaHCO_3_^20^. It is proved by Shaver et al.^22^ that 3:1 ratio of NaHCO_3_ and MgO had best response, thus recommended concentration for dietary buffer supplementation. Meanwhile, the efficiency and mechanisms of buffering agents responsible for alleviating chronic acidosis are variable and often inconsistent^23^. In this study, we hypothesized that BC_0.9%_ could enhance the ruminal fermentation parameters and affect rumen microbiota of Hanwoo steers. This research is a preliminary study on the effect of different levels of buffering agents on ruminal fermentation parameters and rumen bacterial composition. Research on a different level of buffering capacity to enhance ruminal fermentation characteristics and rumen microbiota using a high-concentrate diet has not yet been investigated, hence this study was conducted. For this reason, we used the 3 different buffering capacity concentrations to evaluate their effects on ruminal fermentation characteristics and rumen microbiome. Therefore, in the present work, we investigated the effects of different levels of buffering capacity of buffer agents on in vitro rumen fermentation characteristics, and bacterial community through in vivo trials in high-concentrate fed Hanwoo steers.

## Results

### Effect of different buffering capacities on in vitro rumen fermentation parameters

The buffering capacity of BC_0.9%_ and BC_0.5%_ were significantly greater (*P* < 0.05) after 24 h incubation compared to BC_0.3%_ and CON (Table 1). Both BC_0.9%_ and BC_0.5%_ exhibited significantly higher (*P* < 0.05) buffering capacity. BC_0.9%_ and BC_0.5%_ showed higher (*P* < 0.05) ruminal pH than other treatments throughout the incubation period. Total gas production increased significantly (*P* < 0.05) in BC_0.9%_, BC_0.5%_, and BC_0.3%_ after 12 h, maintaining the CON as the lowest gas produced (182.67 ml, 179.00 ml, 187.00 ml, and 169.00 ml, respectively). The NH_3_-N concentrations for BC_0.9%_ and BC_0.5%_ were significantly higher (*P* < 0.05) than BC_0.3%_ and CON after 3 h incubation. However, no significant differences were observed between treatments during the 6 and 12 h incubation periods. After 24 h incubation, production of NH_3_-N was observed to be higher under BC_0.9%_, followed by BC_0.5%,_ BC_0.3%_, and CON.

**Table 1:** Effect of different buffering capacity concentrations on in vitro rumen fermentation parameters at 3, 6, 12 and 24 h.

Parameters	Time (h)	Treatment^e^				SEM	*P*-value	
		CON	BC_0.3%_	BC_0.5%_	BC_0.9%_		All	Linear
Buffering capacity (meq/l)	3	76.44^d,z^	83.89^c,y^	87.56^b,x^	90.11^a^	0.398	< 0.001	< 0.001
	6	76.45^c,z^	84.17^b,y^	92.89^a,x^	92.89^a^	0.246	< 0.001	< 0.001
	12	85.33^c,y^	87.22^c,y^	96.67^b,x^	99.22^a^	0.469	< 0.001	< 0.001
	24	100.22^c,z^	102.56^b,y^	106.00^a,x^	106.00^a^	0.462	< 0.001	< 0.001
pH	3	6.00^c^	6.03^bc^	6.08^ab^	6.13^a^	0.013	0.012	0.052
	6	5.68^c,z^	5.80^b,y^	5.91^a,x^	5.93^a^	0.014	< 0.001	< 0.001
	12	5.42^c,z^	5.54^b,y^	5.60^a,x^	5.62^a^	0.014	< 0.001	< 0.001
	24	5.14^b,y^	5.15^b,y^	5.22^a,x^	5.24^a^	0.009	0.001	0.003
Total gas (ml)	3	74.67^b,y^	82.00^a,x^	81.67^a,x^	82.00^a^	1.287	0.016	0.004
	6	122.33	124.33	123.00	123.67	1.353	0.777	0.488
	12	169.00^b,y^	187.00^a,x^	179.00^a,x^	182.67^a^	2.492	0.007	0.010
	24	251.67	269.00	256.00	256.67	3.877	0.078	0.195
NH_3_-N (mg/dl)	3	11.26^c,y^	12.24^b,x^	13.05^a,x^	13.39^a^	0.205	0.001	0.003
	6	13.35	13.60	16.09	14.74	0.569	0.178	0.095
	12	13.90^y^	14.95^xy^	16.23^x^	15.84	0.482	0.073	0.010
	24	19.90^c^	20.39^bc^	21.35^ab^	22.43^a^	0.378	0.012	0.052

SEM,  standard error of the mean.

^e^CON (no buffer added); BC_0.3%_ (0.3% buffer); BC_0.5%_ (0.5% buffer); BC_0.9%_ (0.9% buffer).^a–d^Means with different superscripts in a row differ significantly (*P* < 0.05);^x,y,z^ Means within a row indicate linear effect among CON, BC_0.3%_, and BC_0.5%_ (*P* < 0.05).

Significantly higher concentrations (*P* < 0.05) of acetate were observed in BC_0.9%_ at 12 h; however, BC_0.5%_ and BC_0.3%_ obtained higher value (*P* < 0.05) after 24 h (Table 2). Propionate concentration was significantly higher (*P* < 0.05) in BC_0.3%_ and BC_0.9%_ at 6 h. Subsequently, distinct effects of BC_0.3%_, BC_0.5%_, and BC_0.9%_ were observed at 24 h which had significantly higher (*P* < 0.05) propionate concentrations than CON. A similar pattern was noticeable with butyrate at 12 h such that BC_0.3%_, BC_0.5%_, and BC_0.9%_ obtained the higher concentration (*P* < 0.05) compared with CON. During this period, a similar effect can be seen between the 3 treatments; however, no significant effect was noticed on the 24 h observation. Total volatile fatty acid concentrations were higher (*P* < 0.05) in BC_0.3%_, BC_0.5%_ and BC_0.9%_ at 12 to 24 h incubation periods compared to CON. At this time point, treatments BC_0.3%_ and BC_0.5%_ were higher (*P* < 0.05) compared to BC_0.9%_ and CON. Furthermore, there were no treatment effects on acetate to propionate ratio after 24 h incubation. Consequently, increasing the concentration of buffering capacity showed linear effects (*P* < 0.05) on pH, total gas production, NH_3_-N, and at some certain time point of individual VFA.

**Table 2:** Volatile fatty acid production during in vitro rumen fermentation incubated at 3, 6, 12, and 24 h.

Parameters	Time (h)	Treatment^e^				SEM	*P-*value	
		CON	BC_0.3%_	BC_0.5%_	BC_0.9%_		All	Linear
Acetate (mmol/l)	3	100.13	94.81	93.41	90.36	2.505	0.265	0.237
	6	100.03	100.31	99.79	100.39	0.592	0.901	0.775
	12	102.52^b^	104.34^b^	103.36^b^	107.76^a^	0.291	0.001	0.412
	24	103.71^c,y^	112.05^ab,xy^	117.27^a,x^	108.47^bc^	2.182	0.018	0.009
Propionate (mmol/l)	3	27.44	26.51	24.02	25.59	1.395	0.598	0.280
	6	27.81^b^	28.67^a^	27.66^b^	28.37^a^	0.085	0.001	0.421
	12	32.19	32.65	30.59	33.85	0.756	0.227	0.350
	24	35.24^b,y^	40.32^a,xy^	42.31^a,x^	38.09^ab^	1.203	0.045	0.020
Butyrate (mmol/l)	3	17.60	20.15	19.60	19.42	0.501	0.088	0.098
	6	22.23^a,x^	22.78^a,x^	20.95^b,y^	22.81^a^	0.276	0.009	0.006
	12	27.46^b,y^	40.52^a,x^	39.69^a,x^	40.08^a^	1.660	0.007	< 0.001
	24	53.85	54.58	56.43	54.94	0.783	0.327	0.125
Total VFA (mmol/l)	3	145.16	141.47	137.02	135.37	3.381	0.430	0.285
	6	150.07	151.76	148.40	151.57	0.803	0.077	0.196
	12	162.18^b,y^	177.51^a,x^	173.63^a,x^	181.69^a^	2.424	0.005	0.005
	24	192.80^c,y^	206.95^ab,xy^	216.01^a,x^	201.51^bc^	3.447	0.013	0.008
A:P ratio	3	3.74	3.57	3.89	3.53	0.134	0.618	0.670
	6	3.60^ab^	3.50^c^	3.61^a^	3.54^bc^	0.018	0.014	0.719
	12	3.19	3.20	3.41	3.18	0.084	0.465	0.266
	24	2.94	2.78	2.78	2.85	0.069	0.434	0.178

SEM, standard error of the mean.

^e^CON (no buffer added); BC_0.3%_ (0.3% buffer); BC_0.5%_ (0.5% buffer); BC_0.9%_ (0.9% buffer).^a–c^ Means with different superscripts in a row differ significantly (*P* < 0.05);^x,y^ Means within a row indicate linear effect among CON, BC_0.3%_, and BC_0.5%_ (*P* < 0.05).

### Effect of different buffering capacities on rumen fermentation characteristics in Hanwoo steers

The effect of different buffering capacity concentrations on rumen fermentation characteristics of Hanwoo steers in four treatments are presented in supplementary Table 1. Average pH had no significant effects among CON and treatments. However, buffering capacity of BC_0.3%_, BC_0.5%_, and BC_0.9%_ were consistently higher (*P* < 0.05) compared to CON. Other parameters, including NH_3_-N, individual and total VFA were not significantly affected by the treatments during the in vivo trial.

### Bacterial diversity of the rumen contents of Hanwoo steers

**Figure 1:**
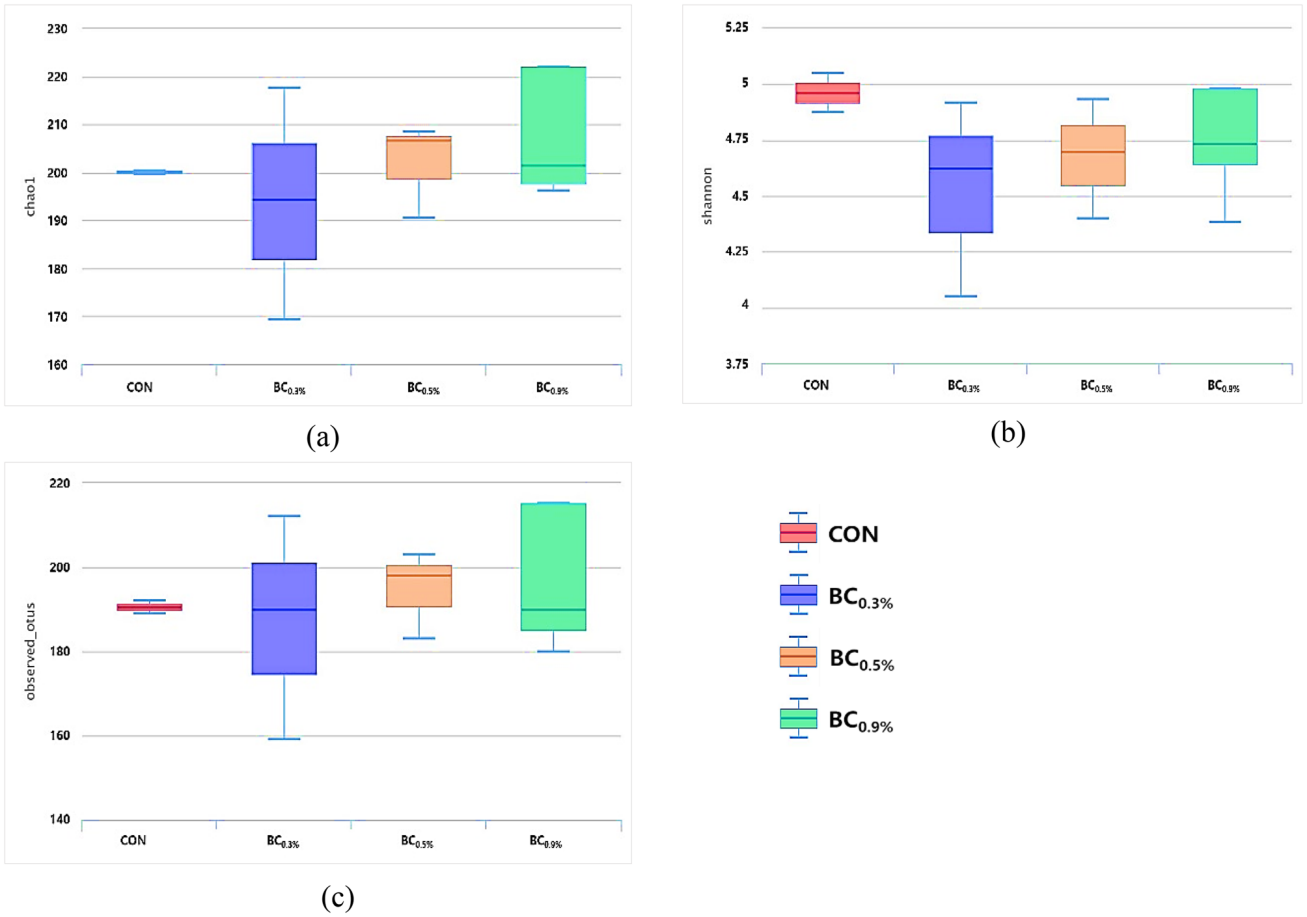
Boxplot representation of alpha diversity indices: (**a**) chao1, (**b**) Shannon, and (**c**) observed OTUs, between treatment groups. Alpha-diversity metrics visualization were done in MetaCOMET^24^ and computed using QIIME^25^. CON (no buffer added); BC_0.3%_ (0.3% buffer); BC_0.5%_ (0.5% buffer); BC_0.9%_ (0.9% buffer).

The boxplot representation of alpha diversity indices is shown in Fig. 1. Alpha diversity indices are composite indices that reflect abundance and consistency. Chao1 which reflect the OTU abundance in the samples showed that BC_0.9%_ was the highest among treatments followed by BC_0.5%_ and the rest of the treatments (Fig. 1a). Shannon index which reflects the diversity of the OTU in samples presented BC_0.9%_ as the most diverse among treatments and BC_0.3%_ being the least (Fig. 1b). Moreover, Fig. 1c shows the boxplot of OTUs of observed species from the samples. The number of OTUs in BC_0.9%_ was higher followed by BC_0.5%_ and the rest of the treatments. Our results showed that the rumen bacterial composition of BC_0.5%_ and BC_0.9%_ had overall higher alpha diversity than other treatment groups, although no significant difference was observed after statistical analysis.

### Effect of treatments on bacterial community composition of Hanwoo steers rumen contents

**Figure 2:**
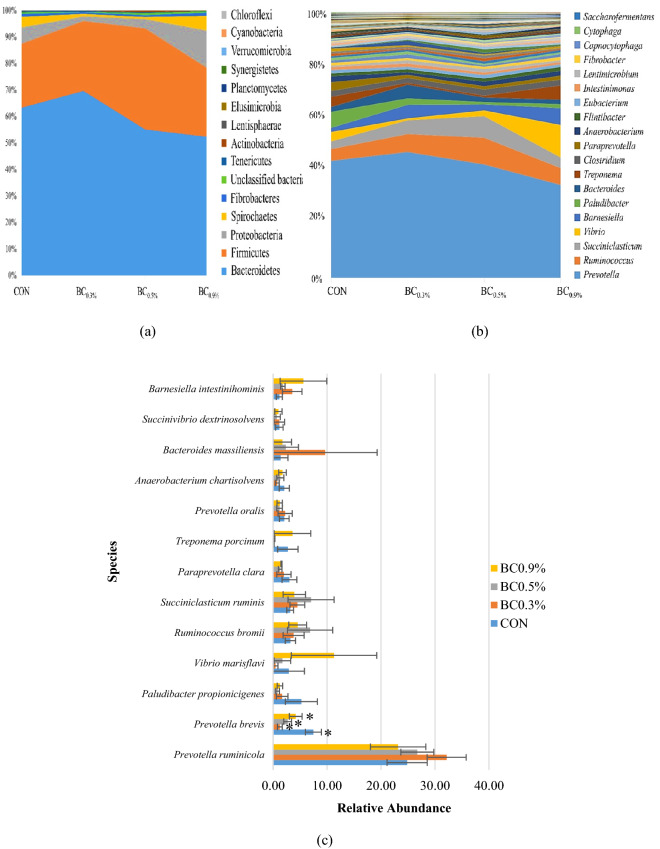
Relative abundance of the observed (**a**) phyla, (**b**) genera, and (**c**) species from the four different treatments. Mean relative abundances of bacterial phyla and genera are presented in supplementary Table 2. Relative abundance was computed using QIIME^25^. CON (no buffer added); BC_0.3%_ (0.3% buffer); BC_0.5%_ (0.5% buffer); BC_0.9%_ (0.9% buffer); asterisk (*): represents significant differences (*P* < 0.05).

Bacterial taxonomic compositions at the phylum, genera, and species level are shown in Fig. 2. Results at the phylum level revealed that 15 bacterial phyla were identified in the rumen digesta samples of Hanwoo steers (Fig. 2a). The majority of the sequences obtained from all treatments belonged to Bacteroidetes followed by Firmicutes. It was noticeable that BC_0.3%_ had the highest abundance of Bacteroidetes (71.90%) and lowest Firmicutes (22.13%). On the contrary, BC_0.9%_ had the lowest abundance of Bacteroidetes (54.19%) among treatments; however, BC_0.5%_ had the highest Firmicutes (33.84%) relative abundance. Proteobacteria and Spirochaetes were abundant under BC_0.9%_ and its abundance reduced both in BC_0.3%_ and BC_0.5%_.

The analysis of genus-level composition revealed 195 genera, of which dominant were presented in Fig. 2b. *Prevotella* was found to be predominant among the genera. The relative abundance of *Ruminococcus* and *Succiniclasticum* were higher in BC0.3% and BC0.5%. Supplementation with BC0.9% increased the abundance of *Vibrio, Barnesiella,* and *Treponema*, whereas it decreased *Paludibacter* and *Bacteroides*. Meanwhile, a noticeable increase in abundance was also observed in *Barnesiella* and *Bacteroides* with the supplementation of BC0.3%. At the species level, *Prevotella ruminicola* was the most abundant among the identified species for all treatments (Fig. 2c). The supplementation of BC_0.3%_ increased its relative abundance. The comparison of single species analyzed through statistical analysis showed a significant effect of the treatments only in the case of *Prevotella brevis.* This species was more abundant in the CON than those supplemented with BC_0.9%_, BC_0.5%_ and BC_0.3%_. Owing to the BC_0.5%_ supplemented in the diet, a decreasing abundance of *Paludibacter propionicigenes* was observed; however, an increase in abundance was notable under CON. Incorporation of BC_0.5%_ increased the microbial population of *Ruminococcus bromii* and *Succiniclasticum ruminis.* Moreover, regardless of the low concentration of BC_0.3%_, supplementation of this treatment increased the abundance of *Bacteroides massiliensis*. Supplementing buffering agents of different buffering capacity concentration may alter the rumen microbiota as what have presented in the result of the present study.

**Figure 3:**
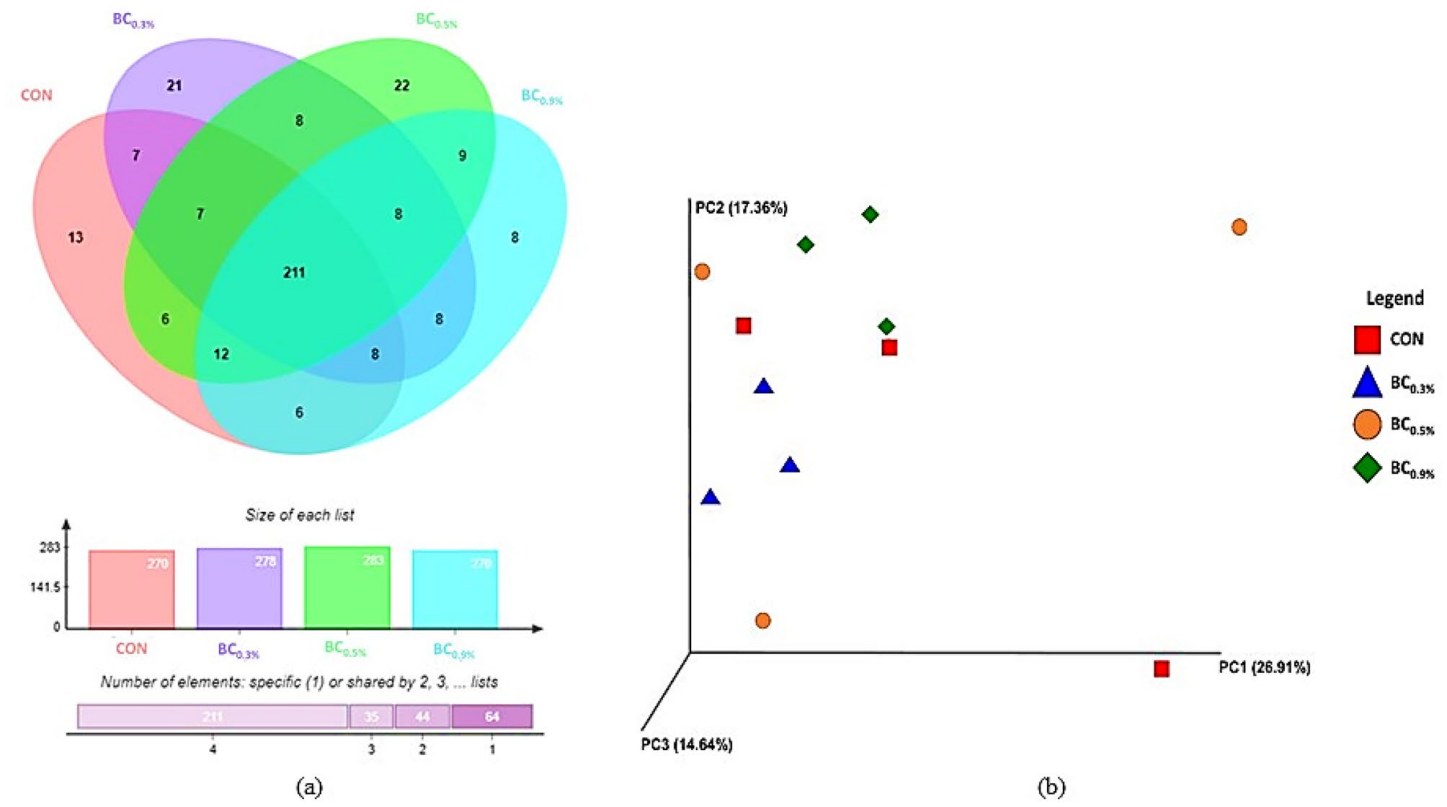
(**a**) Membership-based representation of unique, shared and core bacterial community of rumen microbiome after treatment supplementation with varying level of buffering capacity, and the total size of observed species per treatment. Venn diagram was generated in MetaCOMET^24^ using jvenn^26^. (**b**) Principal Coordinate Analysis (PCoA) of all samples using Bray–Curtis distance derived from the subset of identified OTUs. PCoA plot was generated using EMPeror^27^. CON (no buffer added); BC_0.3%_ (0.3% buffer); BC_0.5%_ (0.5% buffer); BC_0.9%_ (0.9% buffer).

The core, shared and unique bacterial community of observed species of the rumen microbiome after treatment of buffering agents with varying level of buffering capacity is presented in Fig. 3a as Venn diagram. A total of 211 (59.6%) observed species can be found across all the samples (core), 79 (22.32%) for shared by 2 or 3 samples, and 64 (18.08%) are specific and are distributed to the four samples (13 for CON, 21 for BC_0.3%_, 22 for BC_0.5%_, and 8 for BC_0.9%_). The comparison of the bacterial communities by unweighted unifrac diversity principal coordinate analysis (PCoA) is presented in Fig. 3b. The PCoA plots showed close similarity within and between treatments and control, whereas those under BC_0.9%_ and BC_0.3%_ groups showed distinct and spatial separation of bacterial communities.

**Figure 4:**
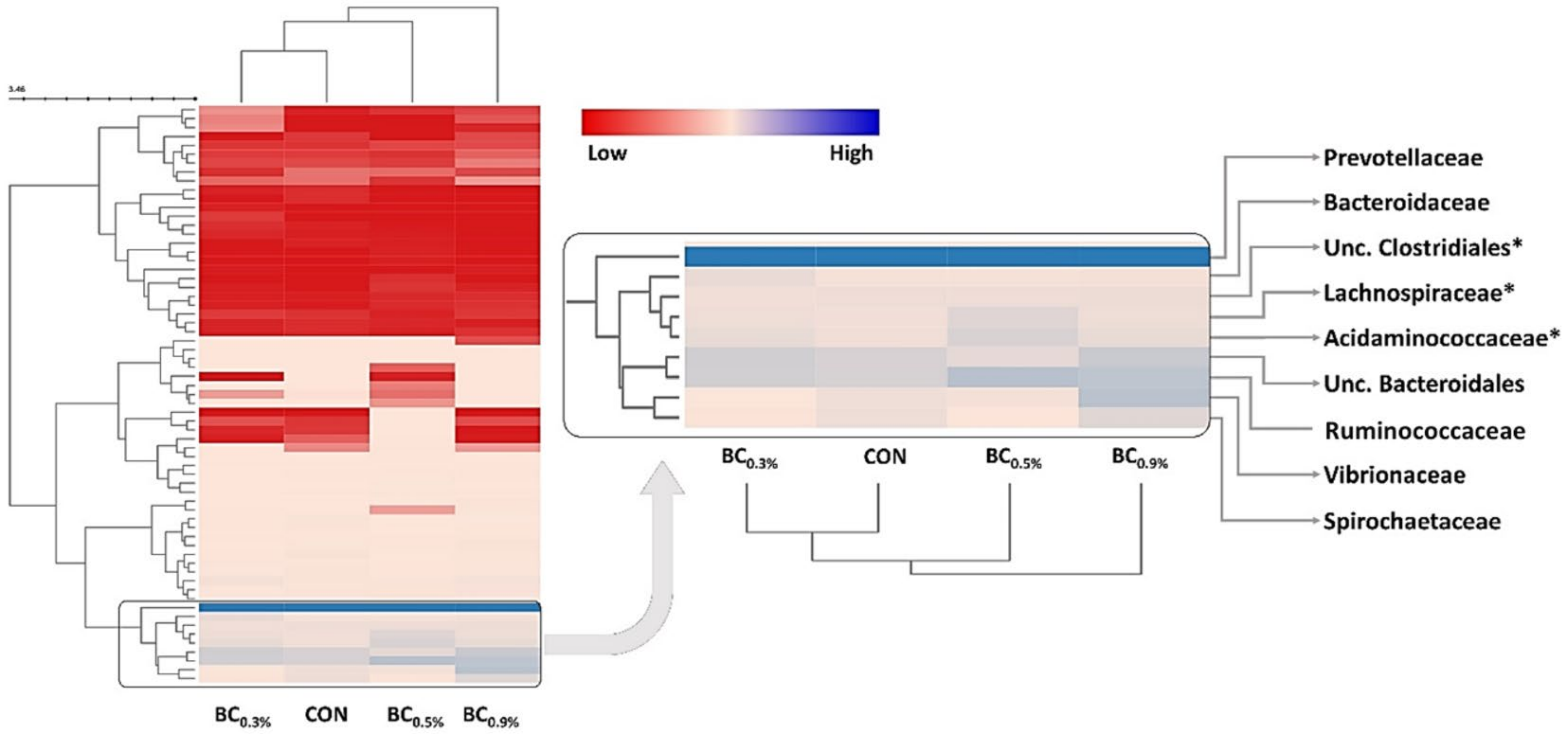
Heatmap presentation of relative abundance of representative families of observed OTUs. Treatments (columns) and families (rows) are clustered using Bray–Curtis dissimilarity test and Ward linkage. Normalized relative abundance are plotted from low (red), mid (peach), and high (blue). Heatmap clustering was generated in MetaCOMET^24^ utilizing the InCHlib application^28^. CON (no buffer added); BC_0.3%_ (0.3% buffer); BC_0.5%_ (0.5% buffer); BC_0.9%_ (0.9% buffer); asterisk (*): represents significant differences (*P* < 0.05).

The normalized data presented in Fig. 4 shows the clustering based on the similarity of relative abundance between representative families of OTUs (row), and treatments (column). The analysis divided the representative families into two major clusters distinguishing families which represents low relative abundance on all treatments (upper cluster in red), and families that have varying relative abundance between treatments (lower cluster, colored from peach to blue). On the cluster presenting varying abundance between treatments, two sub-clusters were also distinguishable; (1) families which represent variation from very low (red) to average (peach) abundance, and (2) cluster representing families which have average to high (blue) abundance. The cluster in the bottom of the heatmap (labelled) contains the families that represent the above average relative abundance. On this cluster, family *Prevotellaceae* had branched out because it presents the highest abundance with very small variations between treatments (*p* = 0.092). Family *Ruminicoccaceae* were also found in all treatments, but varying relative abundance was observed, with BC_0.5%_ presenting the highest. Families *Acidaminococcaceae* and *Lachnospiraceae* were significantly higher (*P* < 0.05) in BC_0.3%_ and BC_0.5%_, respectively. Also, the Unclassified *Clostridiales* had significantly highest (*P* < 0.05) relative abundance in BC_0.5%_. A certain unclassified family under order *Bacteroidales* also showed major abundance especially in BC_0.3%_, while families *Vibrionaceae* and *Spirochaetaceae* were highest in BC_0.9%_. Meanwhile, the normalized data of relative abundance of representative families of observed OTUs have grouped the control sample together with BC_0.3%_ in a single cluster, while BC_0.5%_ and BC_0.9%_ are on their own cluster.

### Monitoring of acidosis

The changes in the 24 h mean ruminal pH monitored for 30 d is presented in supplementary Table 3. During this period of the feeding challenges, mean pH values were > 5.8. Minimum pH was lowest in CON, whereas it was highest in BC_0.5%_. Additionally, BC_0.9%_ had a low minimum pH value second to that of CON. It was noticeable that BC_0.3%_ and BC_0.5%_ had higher minimum and mean pH values compared to BC_0.9%_ and CON. Obtained results indicated that the duration of time where pH was < 5.8 and 5.8 < 6.0 was longer in CON followed by BC_0.9%_ and BC_0.3%_. Meanwhile, BC_0.3%_ also exhibited good results in the duration of time where pH was approximately 6.0 and above; however, BC_0.5%_ had even better effects in the rumen. Based on the data gathered, BC_0.5%_ stabilized the pH of rumen preventing it from becoming acidotic.

## Discussion

One of the major health issues in dairy farming is the sudden decline of ruminal pH that causes reduction of feed intake, problems with digestion, and production losses^10^. Its prevalence adversely affects cattle health resulting to high management cost. Methods of decreasing the rate and incidence of acidosis have already been studied, such as addition of buffering agents and supplementation of commercially available treatments in high-grain diets that presumably reduce acidosis by facilitating intake^29^. Various studies have reported that adding buffer solution, such as sodium bicarbonate and/with magnesium oxide increased dry matter intake when corn silage was the sole or major source of forage in the diet^19^. Moreover, several researches have conducted to control rumen pH through supplementing rations with buffers or neutralizing agents such as sodium bicarbonate or magnesium oxide^30^. Sodium bicarbonate is a naturally secreted buffering agent in the saliva of the cow however when too little saliva is produced, such as during feeding of high-grain rations, supplemental sodium bicarbonate can be of benefit. In addition, this buffer agent is widely used in preventing ruminal acidosis since it provides a natural buffer however, its high solubility limits the buffering activity against acidic condition^20^. Despite its buffering ability, it only functions for a short period of time and because of the high solubility, it is rapidly used by the ruminants. Most studies have suggested that magnesium oxide act either as a neutralizer or buffer in rumen or intestine^31^. It also increases starch digestion in the intestine of animals fed with a high-concentrate diet. This may result in an increase of pH in the small intestines allowing starch-digesting enzymes to become more active^22^. Mao et al.^32^ reported that supplementation of the bicarbonate group had higher pH, total gas production, and total VFA concentration although ammonia–nitrogen concentrations remained unaltered. Addition of combined buffers in high concentrate rations altered rumen pH, liquid turnover, and patterns of rumen fermentation^33^. Consequently, commercial buffer agent (CBA) is developed as a buffer premix and considered as more powerful alternative to sodium bicarbonate. This premix is a mixture of various raw materials, differing in acid-binding capacity and solubility that contained live yeast, which promoted the conversion of lactate to propionate; thus, improving rumen conditions. Research data have shown its efficiency in maintaining the stability of ruminal pH, thus preventing the stimulation of subacute ruminal acidosis^34^. Meanwhile, the results of the present study are in accordance with their experimental output.

The result of the present study showed that BC_0.9%_, as well as the BC_0.5%_, had similar effects on rumen content. These two treatments had significant effects on pH, buffering capacity, and ammonia–nitrogen concentration which only shows that supplementation of buffer agents can enhance ruminal fermentation parameters. An increase in ruminal pH upon supplementation of sodium bicarbonate is a result of dissociation of sodium (Na^+^) and bicarbonate (HCO_3_^−^)^17^. Dijkstra et al.^35^ stated that the interactions between rumen and host and other complex factors that determine ruminal pH might result in wide range differences among animals in terms of rumen pH response to dietary changes. Meanwhile, the results on gas production were supported by the claims of Rauch et al.^36^ and Kang and Wanapat^16^, who stated that supplementation with sodium bicarbonate enhanced gas production. The increase in gas production might be caused by the dissociation of sodium bicarbonate resulting to increase gas volume because of CO_2_ liberation^36^. Also, it might be due to the conversion of some bicarbonate to carbonic acid which soon released as carbon dioxide^17^. NH_3_-N is the vital source of nitrogen for microbial protein synthesis in the rumen^67^. Moreover, obtained data from the present study is in accordance with the results of Le Ruyet and Tucker^21^ on the temporal effects of ruminal buffers in terms of buffering capacity and pH of ruminal fluid from cows fed a high concentration diet. Buffering compounds increased the ruminal fluid buffering value index and were beneficial in preventing postprandial increases in ruminal fluid hydrogen ion concentration. Through this bicarbonate dependent mechanism, the rumen epithelium supplies adequate amount of bicarbonate to the rumen in comparison to the bicarbonate produced during salivation^35^. Thus, this mechanism influences rumen buffering capacity^35,37^. Shaver et al.^22^ also stated that magnesium oxide and sodium bicarbonate were the best rumen buffers, which increased the acetate: propionate ratio and prevented declines in pH. The effect of buffers on VFA in this study was the same as the data obtained by Kang and Wanapat^16^ wherein supplementation with buffering agents increased the total VFA. High ruminal VFA concentration is caused by increased carbohydrate fermentation in the rumen^38^. Although the present study did not show a significant effect on molar concentration of VFA, the noticeable increasing numerical values were observed in buffer-supplemented treatments.

Subsequently, the 16S rRNA gene-based metataxonomic survey of bacterial community composition was identified in the rumen digesta samples of Hanwoo steers. Obtained results at the phylum level were in accordance with the data gathered by Nagata et al.^39^ wherein the relative abundance of Bacteroidetes was higher during the high-concentrate period of the experimental animals. Additionally, Zhao et al.^40^ stated that the microbial community of beef cattle was dominated by Bacteroidetes and Firmicutes at the phylum level regardless of group. An increase in the phylum Bacteroidetes resulted in increased *Prevotella* and repressed Firmicutes, which was attributed to decreasing *Ruminococcaceae*. Dodd et al.^41^ and Naas et al.^42^ indicated that the Bacteroidetes in the rumen represented another numerically dominating phylum that was not associated with cellulose degradation, rather its saccharolytic status is based on limited case studies of noncellulolytic *Prevotella* rumen isolates. Because of the ability of *Prevotella* to use a variety of substrates, it tends to dominate in the rumen under a range of diets^43^. In the present study, *Prevotella ruminicola* appeared to be the predominant species among all treatments. This species constitutes one of the most numerous groups recovered from the rumen and plays important roles in the utilization of polysaccharides of plant origin^44–46^ and the metabolism of peptides and proteins^47–51^. Moreover, the low-relative abundance of *Ruminococcus* (8.93%) in this study was in contrast with the findings obtained by Klieve et al.^11^, who also used a high-grain diet (75% barley) for the animals, although, this genus was identified and largely comprised the cellulolytic bacteria. Meanwhile, Klieve et al.^11^ and Kim et al.^12^ have found that several species of *Ruminococcus* such as *R. bromii* are capable of fermenting starch. This might also explain the abundance of *R. bromii* in the study of Ramos et al.^10^ who also used high-concentrate diet for the animals. High propionate concentration of BC_0.5%_ might be caused by the high relative abundance of *Succiniclasticum ruminis.* This result is in accordance with the previous studies which stated that this species specializes in fermenting and converting succinate to propionate^10,52–54^, which is an important precursor of glucose in ruminants^52^. Ueki et al.^55^ described *Bacteroides massiliensis* as a producer of acetate, propionate, and succinate. We observed a higher abundance of this bacterium among treated groups in a modest level, which could be one of the major contributors on the increase in molar concentrations of VFA’s on in vivo study. The abundance of *Paludibacter propionicigenes* might be due to its description as a sugars utilizer and a producer of acetate and propionate, an end product of fermentation^56^.

Acidosis was defined as impaired ruminal health accompanied by a reversible ruminal pH depression^11,57–60^. Ruminal microbes convert carbohydrates to short-chain fatty acids at a rate that exceeds the rumen’s absorptive, buffering, and outflow capacity causing a rapid decrease in ruminal pH^61^. Meanwhile, Zamarreño et al.^23^ stated that the use of sodium bicarbonate and magnesium oxide or even mixed antacids were recommended for satisfactory results. They concluded that the increase in buffering capacity and increase in acid consuming capacity contributed to the correction of animal acidosis.

In conclusion, supplementation of BC_0.5%_ and BC_0.9%_ buffer agents increased the buffering capacity, enhanced the rumen fermentation, and alters rumen microbiome, which is an important factor contributing positively to the correction of animal acidosis during a high-concentrate diet. Furthermore, this study also considers other mechanisms such as salivation of the animals and VFA absorption through epithelium, which might play an equal relevant roles in altering rumen microbiota.

## Methods

### Animal care and ethics approval

Animals used in this experiment and all experimental protocols were reviewed and approved by the Sunchon National University Animal Research Ethics Committee (SCNU IACUC, approval number: SCNU IACUC-2018-01). All experiments were performed in accordance with the guidelines and regulation set by the governing body, and carried out in compliance with the Animal Research Reporting In Vivo Experiments (ARRIVE) guidelines.

### Animals, rumen fluid collection and in vitro rumen fermentation

Three ruminally cannulated Hanwoo steers (500 ± 47 kg body weight; 20 mos. of age) were used to provide ruminal fluid for in vitro rumen fermentation. The animals were fed twice daily with concentrate feed and kleingrass (*Panicum coloratum* L.). Ruminal contents were collected before morning feeding. Samples were squeezed and strained through four layers of surgical gauze and pooled in an amber bottle with an oxygen-free headspace, which was subsequently capped after collection. Collected samples were immediately transported to the laboratory while being maintaining at a temperature of 39 °C^62^.

Seventy milliliters of rumen fluid were dispensed into serum bottles containing each treatment and 2.5 g dry matter of ground corn grain served as substrate, mixed, and flushed with CO_2_^63^. Samples were in triplicate and incubated at 39 °C for 3, 6, 12, and 24 h while shaking horizontally at 100 rpm, as described by Hattori and Matsui^64^. The buffering agents used in treatments are composed of calcium carbonate, magnesium oxide, sodium carbonate, and calcified seaweed (Rupromin Balance™, Rotterdam, Netherlands). Treatments consisted of CON (negative control, no buffer added), BC_0.3%_ (low buffering capacity, 0.3% buffering agent), BC_0.5%_ (medium buffering capacity, 0.5% buffering agent), and BC_0.9%_ (high buffering capacity, 0.9% buffering agent). The buffering agents and the concentrate given to experimental animals were supplied by Purina® Cargill, Korea. The ingredients and chemical composition of the experimental concentrate offered are presented in supplementary Table 4. Treatments were initially tested for determining their neutralizing (NC) and buffering capacity (BC) through titration using 2 N acetic acid from its initial pH to 6.50, and 5.50, respectively (Supplementary Table 5). The buffering agents used in every treatment are in powdered form.

### Analyses of in vitro rumen fermentation parameters and buffering capacity

Ruminal fermentation parameters were monitored at the end of each incubation time period. Total gas production was measured from each serum bottle after the incubation time using a pressure meter (Laurel Electronics, Inc., Costa Mesa, Calif., USA). Consequently, a needle channel connected to the machine was extended into the sealed fermentation bottle for measuring positive pressure created by the gas build up inside the bottle. A gas flow regulator was then opened to allow gas flow inside a syringe barrel and the plunger was subsequently pulled gradually until the pressure reading on the machine display was zero. The volume of gas trapped inside the barrel was recorded as the total gas produced^62,65^.

The pH value was determined using a pH meter (Metler Toledo, Germany) after uncapping each serum bottle. Samples of fermenta were also collected into two 1.5 ml microcentrifuge tubes and stored at − 80 °C prior to ammonia–nitrogen and VFA analyses. Frozen samples were thawed at room temperature; after which, they were centrifuged for 10 min at 13,000 rpm at 4 °C using a Micro 17TR centrifuge (Hanil Science Industrial, Korea). The resulting supernatant was used for ammonia–nitrogen and VFA concentration analyses. Ammonia–nitrogen concentration was measured according to the colorimetric method developed by Chaney and Marbach^66^ using a Libra S22 spectrophotometer (Biochrom Ltd., CB40FJ, England) at an absorbance of 630 nm. Analysis of volatile fatty acid concentration was done using high-performance liquid chromatography (Agilent Technologies 1200 series, Tokyo, Japan) with a UV detector set at 210 nm and 220 nm. Samples were isocratically eluted with 0.0085N H_2_SO_4_ at a flow rate of 0.6 ml/min and a column temperature of 35 °C.

Ruminal fluid pH was recorded following 1 min of equilibration. Buffering capacity, defined as the resistance to change in pH from pH 7 to 5, was determined by titrating a 30 ml aliquot of ruminal fluid with continuous stirring from its initial pH to pH 5 with 1 N HCl and titrating an additional 30 ml aliquot from its initial pH to a pH of 7 with 1 N NaOH. If the initial pH was higher than 7, only the volume of acid required to reduce the pH from 7 to 5 was recorded. Buffering capacity was converted to milliequivalents per liter as follows: BC = [(milliliters of 1 N HCl) + (milliliters of 1 N NaOH)] × 10^3^/30^21^.

### Analysis of rumen fermentation characteristics in Hanwoo steers

In vivo experiment was conducted using four non-cannulated Hanwoo steers (765 ± 60 kg body weight; 24 mos. of age) in a 4 × 4 Latin square design to assess the effects of treatments on rumen fermentation characteristics and ruminal bacterial composition and diversity of the experimental animals for four months. The feeding trial was conducted with 4 treatments comprised of CON which served as the non-buffer supplemented group, BC_0.3%_, BC_0.5%_, and BC_0.9%_. Each experimental period lasted for a month with 10 days washing (animals were fed with control), 20 days adaptation period (animals were fed with different treatments or control), and rumen sampling collection at the end of the period.

### Experimental design and animal management

The Hanwoo steers were fed daily of 2:8 forage (2 kg) and concentrate (2 kg) ratio in 2 equal portions at 9:00 AM and 4:00 PM. Animals in all treatments received the same vaccinations, medications, and were under the same management programs unless otherwise stated. Steers were confined in free-stall barns and had free access to water and exercise lots.

Rumen fluid samples were collected before morning feeding using an oral stomach tube on the 30th day right before transitioning to the next feeding trial for the analysis of ruminal fermentation parameters. These parameters which include rumen pH, total gas, NH_3_-N, and VFAs were all evaluated using the same protocol as used in the in vitro experiment. However, rumen pH change in every experimental period of about 30 days was monitored using eCow (hathor.ecow.co.uk). The eCow system monitors the rumen pH every minute and averaged every 15 min. It was done basically to monitor the occurrence of acidosis through a pH value of < 5.8. This threshold value was used because it is harmful to ruminal cellulolytic bacteria^75^.

### 16S ribosomal RNA gene amplicon sequencing and metataxonomic analyses

Samples obtained from each treatment were sent to Macrogen, Korea for DNA extraction, 16S rRNA gene sequencing and microbiome analysis. Ruminal fluid samples were transported with dry ice to protect the samples from degradation due to extended shipping time or elevated temperatures. In brief, DNA was extracted using DNeasy Power Soil Kit (Qiagen, Hilden, Germany) according to the manufacturer’s instructions. The extracted DNA was quantified using Quant-IT PicoGreen (Invitrogen). The sequencing libraries were prepared according to the Illumina 16S Metagenomic Sequencing Library protocols to amplify the V3 and V4 region. The input gDNA was PCR amplified with 1 × reaction buffer, 1 nM of dNTP mix, 500 nM each of the universal F/R PCR primer, and 2.5 U of Herculase II fusion DNA polymerase (Agilent Technologies, Santa Clara, CA). The cycle condition for 1st PCR was 3 min at 95 °C for heat activation, and 25 cycles of 30 s at 95 °C, 30 s at 55 °C and 30 s at 72 °C, followed by a 5-min final extension at 72 °C. The universal primer pair with Illumina adapter overhang sequences used for the first amplification was V3-F (5′-TCG TCG GCA GCG TCA GAT GTG TAT AAG AGA CAG CCT ACG GGN GGC WGC AG-3′) and V4-R (5′- GTC TCG TGG GCT CGG AGA TGT GTA TAA GAG ACA GGA CTA CHV GGG TAT CTA ATC C-3′). The 1st PCR product was purified with AMPure beads (Agencourt Bioscience, Beverly, MA). Following purification, the 2 μL of 1st PCR product was PCR amplified for final library construction containing the index using NexteraXT Indexed Primer. The cycle condition for the 2nd PCR was the same as the 1st PCR condition except for 10 additional cycles. The PCR product was purified with AMPure beads. The final purified product is then quantified using qPCR according to the qPCR Quantification Protocol Guide (KAPA Library Quantification kits for Illumina Sequencing platforms) and qualified using the TapeStation D1000 ScreenTape (Agilent Technologies, Waldbronn, Germany).

Sequencing was done using the Illumina Miseq (Illumina Inc., San Diego, CA, USA) platform. The raw data files (fastq) containing the sequenced paired-end (PE) reads were obtained using the bcls2fastq package (Illumina Inc., San Diego, CA, USA) from the base call binary data produced by real-time analysis. The PE raw reads were filtered from adapter sequences using Scythe (v0.994)^68^ and Sickle^69^ programs then assembled using Fast Length Adjustment of Short Reads (FLASH 1.2.11)^70^. Assembled reads were quality filtered and trimmed for short and extra-long reads, and duplicate reads were removed, then clustered at 100% identity using CD-HIT-OTU^71^. Chimeric reads were identified and the initial clusters were recruited to primary clusters. Then, noise filtering was done and the remaining non-chimeric clusters were binned to operational taxonomic units (OTU) following a greedy algorithm with a cut-off value of 97% species level identity using CD-HIT-OTU^71^. Representative sequences from the clustered OTU were taxonomically assigned using Quantitative Insights Into Microbial Ecology (QIIME Version 1)^25^ from the NCBI 16S rRNA gene database, and the taxonomy composition was generated using QIIME-UCLUST^72^. The produced bacterial taxonomy and composition data were used to generate a biological information matrix (BIOM)^73^ in Mothur^74^. The generated BIOM file were used to visualize the alpha and beta diversity indices, and the bacterial composition using programs utilized by Metagenomics Core Microbiome Exploration Tool (MetaCOMET)^24^.

### Statistical analysis

Data analysis was performed using Statistical Analysis Systems (SAS) version 9.1 (SAS Institute Inc., Cary, NC). The data of rumen fermentation, alpha diversity indices and relative abundance of individual taxa of rumen microbiota were statistically evaluated using Proc general linear model (GLM) for a completely randomized design. All treatments in the in vitro experiment were conducted in triplicate and Duncan’s Multiple Range Test (DMRT) was used to identify differences between specific treatments. The linear effects of different buffering capacity concentrations were analyzed using orthogonal polynomial coefficients to describe the functional relationships among the control and treatment groups. A *P* < 0.05 was considered indicative of significant differences.

## Supplementary Information

Supplementary Tables.
