# Supplementary material for: Increasing buffering capacity enhances rumen fermentation characteristics and alters rumen microbiota composition of high-concentrate fed Hanwoo steers
Source: Sci Rep. 2022 Dec 1;12:20739. doi: 10.1038/s41598-022-24777-3 (PMC9715728; doi:10.1038/s41598-022-24777-3)
Supplement: Supplementary file 1 — Supplementary Tables. [file 41598_2022_24777_MOESM1_ESM.docx]

| **Parameters** | **Treatment ^c^** | | | | **SEM** | ***P-*value** | |
| --- | --- | --- | --- | --- | --- | --- | --- |
|  | **CON** | **BC_0.3%_** | **BC_0.5%_** | **BC_0.9%_** |  | **All** | **Linear** |
| Average pH | 6.28 | 6.56 | 6.77 | 6.48 | 0.146 | 0.226 | 0.528 |
| Buffering capacity (meq/L) | 83.47^b,y^ | 99.53^a,x^ | 101.00^a,x^ | 94.80^a^ | 3.058 | 0.015 | 0.004 |
| NH_3_-N (mg/dL) | 4.84 | 4.26 | 5.40 | 4.59 | 1.260 | 0.945 | 0.794 |
| Total VFA (mmol/L) | 47.24 | 45.29 | 54.16 | 55.87 | 5.234 | 0.632 | 0.563 |
| Acetate (mmol/L) | 29.67 | 27.28 | 30.70 | 35.56 | 2.421 | 0.532 | 0.890 |
| Propionate (mmol/L) | 11.30 | 9.26 | 14.37 | 12.75 | 2.190 | 0.412 | 0.380 |
| Butyrate (mmol/L) | 6.28 | 8.76 | 9.08 | 7.56 | 1.556 | 0.652 | 0.299 |
| A:P ratio | 2.81 | 3.10 | 2.13 | 2.91 | 0.451 | 0.431 | 0.370 |

**Supplementary Table 1.** Effect of different buffering capacity concentrations on rumen fermentation characteristics in Hanwoo steers. ^c^ CON (no buffer added); BC_0.3%_ (0.3% buffer); BC_0.5%_ (0.5% buffer); BC_0.9%_ (0.9% buffer). SEM = standard error of the mean. ^a-b^ Means with different superscripts in a row differ significantly (*P* < 0.05); ^x,y^ Means within a row indicate linear effect among CON, BC_0.3%_, and BC_0.5%_ (*P* < 0.05).

| **Taxonomic level** | | **Treatment** | | | |
| --- | --- | --- | --- | --- | --- |
|  |  | **CON** | **BC_0.3%_** | **BC_0.5%_** | **BC_0.9%_** |
| Phyla | Bacteroidetes | 63.29 | 82.81 | 70.02 | 55.35 |
|  | Firmicutes | 24.03 | 31.32 | 48.38 | 27.41 |
|  | Proteobacteria | 6.31 | 2.23 | 4.69 | 15.06 |
|  | Spirochaetes | 3.90 | 1.01 | 0.78 | 5.76 |
|  | Fibrobacteres | 1.15 | 0.89 | 0.97 | 1.39 |
|  | Cyanobacteria | 0.60 | 0.10 | 1.08 | 0.43 |
|  | Tenericutes | 0.36 | 0.33 | 0.34 | 0.15 |
|  | Actinobacteria | 0.05 | 0.10 | 0.61 | 0.07 |
|  | Lentisphaerae | 0.22 | 0.10 | 0.14 | 0.08 |
|  | Elusimicrobia | 0.05 | 0.02 | 0.06 | 0.04 |
|  | Planctomycetes | 0.02 | 0.02 | 0.02 | 0.03 |
|  | Synergistetes | 0.01 | 0.01 | 0.02 | 0.02 |
|  | Verrucomicrobia | 0.00 | 0.05 | 0.00 | 0.00 |
|  | Cyanobacteria | 0.00 | 0.00 | 0.02 | 0.00 |
|  | Chloroflexi | 0.00 | 0.01 | 0.00 | 0.00 |
| Genera | *Prevotella* | 44.11 | 47.42 | 42.70 | 35.06 |
|  | *Ruminococcus* | 4.35 | 6.65 | 10.08 | 6.31 |
|  | *Succiniclasticum* | 2.99 | 5.31 | 8.13 | 3.91 |
|  | *Vibrio* | 3.52 | 0.68 | 2.17 | 12.27 |
|  | *Barnesiella* | 1.46 | 5.10 | 2.13 | 6.35 |
|  | *Paludibacter* | 6.11 | 2.48 | 1.03 | 1.50 |
|  | *Bacteroides* | 1.77 | 5.00 | 1.71 | 1.65 |
|  | *Treponema* | 3.86 | 0.74 | 0.61 | 5.42 |
|  | *Clostridium* | 2.26 | 1.76 | 2.39 | 2.12 |
|  | *Paraprevotella* | 3.29 | 1.23 | 1.21 | 1.56 |
|  | *Anaerobacterium* | 2.10 | 0.96 | 1.61 | 1.83 |
|  | *Flintibacter* | 1.18 | 1.15 | 1.27 | 1.50 |
|  | *Eubacterium* | 1.24 | 0.95 | 1.32 | 0.93 |
|  | *Intestinimonas* | 1.06 | 1.27 | 0.86 | 0.89 |
|  | *Lentimicrobium* | 0.51 | 1.28 | 1.47 | 0.61 |
|  | *Fibrobacter* | 1.15 | 0.75 | 0.76 | 1.32 |
|  | *Capnocytophaga* | 0.83 | 1.40 | 0.60 | 1.04 |
|  | *Cytophaga* | 0.96 | 1.10 | 0.48 | 0.71 |
|  | *Saccharofermentans* | 0.67 | 0.68 | 0.95 | 0.78 |

**Supplementary Table 2.** Relative abundance (%) of major phyla and genera in the rumen sample of high-concentrate fed Hanwoo steers. CON (no buffer added); BC_0.3%_ (0.3% buffer); BC_0.5%_ (0.5% buffer); BC_0.9%_ (0.9% buffer)

| **Item** | **Treatment ^a^** | | | |
| --- | --- | --- | --- | --- |
|  | **CON** | **BC_0.3%_** | **BC_0.5%_** | **BC_0.9%_** |
| *24 h mean ruminal pH* |  |  |  |  |
| Minimum | 5.28 | 5.72 | 5.83 | 5.40 |
| Mean | 6.36 | 6.56 | 6.47 | 6.22 |
| *Duration of ruminal pH* |  |  |  |  |
| pH <5.8, min/d | 66.29 | 1.45 | 0.00 | 13.06 |
| pH 5.8 < 6.0, min/d | 90.97 | 21.77 | 10.16 | 106.45 |
| pH 6.0 and above, min/d | 1214.52 | 1296.77 | 1202.42 | 999.68 |

**Supplementary Table 3.** Changes in the 24 h mean ruminal pH, duration of time where pH was <5.8 monitored for 30 d challenge diet. ^a^ CON (no buffer added); BC_0.3%_ (0.3% buffer); BC_0.5%_ (0.5% buffer); BC_0.9%_ (0.9% buffer).

| **Ingredients ^a^** | **Percentage (%)** |
| --- | --- |
| Corn fine | 31.17 |
| Corn gluten feed | 21.00 |
| Soy hulls | 13.00 |
| Wheat fine | 10.00 |
| Rice bran | 5.00 |
| Wheat flour | 5.32 |
| DDGS | 3.40 |
| Molasses | 3.00 |
| Palm kernel meal | 2.14 |
| Limestone fine | 1.90 |
| Palm kernel meal (Solvent) | 1.80 |
| CMS | 1.50 |
| Brown rice | 0.45 |
| Salt | 0.17 |
| Mineral/Vitamin premix^1)^ | 0.15 |
| Total | 100.00 |
| Calculated nutrients, as fed |  |
| Crude Protein | 13.03 |
| Crude Fat | 3.84 |
| Ash | 5.77 |
| NDF | 23.44 |
| Moisture | 11.50 |
| Crude Fiber | 8.65 |
| Calcium | 1.00 |
| Phosphorus | 0.48 |
| Ca/P | 2.09 |
| Sulfur | 0.20 |
| Potassium | 0.84 |
| Magnesium | 0.24 |
| Sodium | 0.21 |
| TDN | 76.44 |

**Supplementary Table 4.** Ingredients and chemical composition of experimental concentrate. ^a^ DDGS = dried distillers’ grains with solubles; CMS = condensed molasses solubles; NDF = neutral detergent fiber; TDN = total digestible nutrients. ^1)^ Mineral & vitamin premix contained vit. A 2,650,000 IU, vit. D_3_ 530,000 IU, vit. E 1,050 IU, niacin 10,000 mg, Mn 4,400 mg, Zn 4,400 mg, Fe 13,200 mg, Cu 2,200 mg, iodine 440 mg, and Co, 440 mg/kg of Grobic-DC provided from Bayer Health Care (Leverkusen, Germany).

| **Parameters** | **Sample buffers ^d^** | | | **SEM** | ***P-*value** | |
| --- | --- | --- | --- | --- | --- | --- |
|  | **BC_0.3%_** | **BC_0.5%_** | **BC_0.9%_** |  | **All** | **Linear** |
| Initial pH | 6.94^a^ | 7.09^b^ | 7.42^c^ | 0.007 | <0.001 | <0.001 |
| Neutralizing capacity (mmol/L) | 0.03^a^ | 0.06^b^ | 0.16^c^ | 0.001 | <0.001 | <0.001 |
| Buffering capacity (mmol/L) | 0.16^a^ | 0.30^b^ | 0.43^c^ | 0.003 | <0.001 | <0.001 |

**Supplementary Table 5.** Titration results to determine the neutralizing and buffering capacity of sample buffers used in treatments. ^d^ BC_0.3%_ (0.3% buffer); BC_0.5%_ (0.5% buffer); BC_0.9%_ (0.9% buffer). ^a,b,c^ Within row indicate linear effect (*P* < 0.05).
